# Mobile Electronic Devices as Means of Facilitating Patient Activation and Health Professional Empowerment Related to Information Seeking on Chronic Conditions and Medications: Qualitative Study

**DOI:** 10.2196/26300

**Published:** 2021-08-11

**Authors:** Hyllore Imeri, Shane Desselle, Dardan Hetemi, Kreshnik Hoti

**Affiliations:** 1 Division of Pharmacy Faculty of Medicine University of Prishtina Pristina Kosovo; 2 Department of Pharmacy University of Mississippi Oxford, MS United States; 3 Department of Pharmacy Touro University California Vallejo, CA United States

**Keywords:** patient activation, mobile electronic devices, health professionals, chronic conditions, medications

## Abstract

**Background:**

Patient activation has an impact on the management of patients’ health, clinical outcomes, and treatment costs. Mobile electronic devices (MEDs) have shown the potential to engage patients in wellness behavior. Furthermore, the potentially positive role of MEDs is evident in supporting health professionals in their practice.

**Objective:**

This study aims to explore the impact of MEDs on patient activation to search for information on chronic conditions and medications and the impact of MEDs on the empowerment of health professionals or future health professionals.

**Methods:**

We conducted 6 focus groups—2 with health sciences students, 2 with health professionals, and 2 with hospitalized patients with chronic conditions. A protocol comprising eight questions was used to guide discussions. Audio-recorded data were transcribed verbatim and analyzed thematically; a ranking system was used to analyze the relevance of identified themes and subthemes, using a coding system depicted by the + symbol, to indicate different relevance levels.

**Results:**

Our results suggest that MEDs can positively affect patient activation to search for chronic conditions and medication information by facilitating patients’ information-seeking behavior. Key drivers leading to patients’ activation to seek information related to chronic conditions and medications through MEDs were the accessibility and abundance of available and detailed information, reduced search time, information updates, and convenience in finding information at any time and place. The lack of accurate information in one’s native language, access to incorrect information, and limited access to the internet were key obstacles to seeking information related to chronic conditions and medications via MEDs. In addition, findings of this study suggest that MEDs in general and mobile apps, in particular, may have a positive impact on the work routine of health care professionals as they enable them to make quicker decisions by accessing the required information faster, thus improving practice efficiency. Furthermore, the appropriate usage of MEDs by patients for seeking information about their chronic conditions and medications may positively impact the physician-patient relationship. All focus groups recognized the questionable reliability of health information on the internet and its potential negative effects on patients. Therefore, our findings suggest the need for an additional role of health professionals in assisting patients in using MEDs to search for health and medication information, such as providing reliable websites and mobile apps where patients can safely search for health-related information on the web.

**Conclusions:**

The use of MEDs may help activate patients to seek chronic conditions and medication-related information, potentially leading to better management of their chronic conditions and medications. Our findings also highlight the positive impact MEDs may have on empowering health professionals in their practice and the need for health professionals to help patients through specific education that addresses MEDs utilization for chronic conditions and medication information seeking.

## Introduction

### Background

Patient activation is a behavioral concept defined as “the individual’s knowledge, skills and belief in managing his/her health and healthcare” [1]. Patient activation enables an understanding of why some patients engage and are actively involved in their health whereas others are not. Considering the relevance of patients’ roles, health care settings have moved to a more patient-centered model, where patients are encouraged to be effective managers of their health care [2,3]. A higher patient activation level is associated with a wide range of better health outcomes [4], health-related behaviors, and health care costs [5]. In addition, although nearly half of patients assess information about their medical condition or treatments on the web [6], evidence suggests that activated persons are more likely to use web-based health information [7].

Although there have been rapid developments in mobile health (mHealth) apps, their effects on improving health and health care remain unclear [8,9]. However, the role of mobile electronic devices (MEDs) concerning patient engagement and facilitation of communication between patients and health professionals has been recognized. In this regard, several programs using mobile devices to engage and educate patients have been designed [8,10]. Evidence suggests that mobile phone apps can facilitate medication adherence by using functions such as reminder alerts and providing access to drug information [11]. Furthermore, many mobile apps to support health care professionals during their practice have been developed [12-14], thus promoting personalized and efficient health care. Therefore, the potential of MEDs, including laptops, tablets, and especially mobile phones, related to facilitating activation to navigate health information on the web is expected to be highly advantageous to not only patients but also health professionals.

Patient activation levels may increase with time, making patient activation an important focus of interventions to improve health behaviors and outcomes [6,7]. There are four patient activation levels that patients go through as they become more activated, from being disengaged and overwhelmed to maintaining positive health-related behaviors and pushing further [1]. Providing health information to patients is one of the initial steps of engagement in health care self-management. Patient education through health-related informative letters has been found to drive patient activation in patients with chronic conditions such as hypertension, suggesting a significant role of health information in patient activation [15]. However, considering the increased tendency to seek health information on the web [6] and the high rates of MED ownership [16], a potential role of MEDs in facilitating the process of patient education, and consequently the overall patient activation, could be suggested.

### Objectives

Currently, there is limited evidence exploring how MEDs could facilitate the activation of patients seeking information on the web about their health and medications and the acceptance of MEDs on health professionals’ work. This study aims to explore (1) the role of MEDs on patient activation toward seeking information about chronic conditions and medications and (2) the impact of MEDs on health professionals or future health professionals’ empowerment.

## Methods

### Study Design

This qualitative study used focus groups (FGs) as a data collection method, which allowed us to explore various perspectives. Approvals to conduct the study and access to patients were provided by the ethics committee of the Faculty of Medicine, University of Pristina, and the University Clinical Center of Kosovo (UCCK) office for personal data protection, respectively.

### Study Setting and FG Participants

In total, 6 FGs were organized with 4 to 7 participants. These numbers were chosen based on the literature suggesting that themes saturation can be achieved on this basis [17]. In particular, saturation was sought despite seeking input from various groups involved in the medication use process, including patients, health professionals, and student health professionals. To examine the role of MEDs in patient activation toward health information seeking from various perspectives, we conducted FGs with patients, health professionals, and future health professionals. Health sciences students were selected for FG inclusion because of their likelihood of being well-versed and high MEDs users, thus presenting the potential to better understand the acceptance of MEDs from future health professionals. Of the 6 FGs, 2 (33%) were conducted with health science students at the University of Pristina, 2 (33%) with health professionals working at the UCCK, and 2 (33%) with patients with chronic conditions who were hospitalized at the UCCK and receiving more than one medication. FGs were organized on the premises of the UCCK and the Faculty of Medicine, University of Pristina.

### Recruitment

A purposive selection strategy was used, targeting health professional staff of the UCCK, patients with chronic conditions receiving health care treatment at the UCCK, and health sciences students. The study’s setting in Kosovo presents an ideal environment in which to conduct the study, given the high use of internet technology and connectivity of Kosovo’s population. However, there is lack of data that would suggest that Kosovo’s residents leveraged this use into active involvement into technologies that potentially assists them with management of their health conditions or seeking information for health maintenance purposes. Students were recruited using snowball sampling, whereas health professional staff and patients were approached by the facilitator of FGs and the principal author on the UCCK premises and invited to participate in the study. Before inviting patients and health professionals to participate in the study, researchers informed the respective clinics’ chiefs of staff. Patients were approached during the optimal time of the day after the physicians’ consultations, and FGs were conducted on the same day. Health professionals chose their optimal time and date, which was before starting their shifts or during breaks. Potential subjects who agreed to participate also received an information letter and a letter of consent as an invitation to be a participant in the FG. The design of the FG structure and protocol was based on data from previous studies [18].

### FG Discussion Guide

The development of the question protocol for this study considered the concept of patient activation and the utility of MEDs in seeking health information on the web relevant to patients’ health care management. In addition, the researchers who conducted the FGs clarified the definition of MEDs verbally as part of the opening statements by taking examples of mobile phones, tablets, and laptops. The question protocol consisted of an opening question, four transitory questions, and three key questions. The opening question was related to searching for health and medication information and participants’ access to MED use. Transitory questions were related to difficulties in seeking information, factors that would help overcome these obstacles, and patient views on health professionals’ roles. Key questions were related to the potential drivers leading to patient activation to seek information on chronic conditions and medications and the role of MEDs during this process.

The FGs were conducted by a facilitator. The principal author also attended the meetings, took notes, and audio recorded to further facilitate data analysis and mitigate against the potential bias introduced by the facilitator. All FGs were conducted in the native language of the participants, which is Albanian, and were held between May and June 2018. Audio-recorded data from FG meetings were transcribed verbatim into Microsoft Word version 2013 and translated and reviewed by 3 researchers.

### Qualitative Analysis

Data were analyzed thematically in Microsoft Word, using the open, axial, and selective coding strategy [19], initially by one of the researchers of this study, and a second analysis and review were conducted by the other researcher of the study. A ranking system was used to analyze the relevance of the identified themes and subthemes. This approach has been previously reported in the literature [20]. If a similar comment was repeated for a given issue, the + symbol was used. If a comment was repeated only within 1 FG, it was marked +, if a comment was repeated in 2 to 3 FGs, it was marked as ++, and when a comment was repeated in all FGs, it was marked with +++, where + indicates low relevance, ++ indicates average relevance, and +++ indicates high relevance.

## Results

### Overview

A total of 6 FGs involving 31 participants were conducted on the UCCK and Faculty of Medicine premises in Pristina, Kosovo. The 2 FGs with health care professionals were conducted with the health care professional staff of the Clinic of Nephrology and the Infectious Diseases Clinic at the UCCK, 4 and 7 participants, respectively, with ages ranging from 30 to 55 years, of which 73% (8/11) were female. The 2 FGs with patients were conducted with patients admitted to either the Dermatology Clinic (UCCK) or Clinic of Hematology (UCCK), with 4 participants per FG. Patient age ranged from 45 to 75 years, of which 63% (5/8) were women. In addition, a total of 12 students agreed to participate. They were divided into 2 FGs, each composed of 6 participants, with ages ranging from 20 to 25 years, and equal participation of both men and women. The duration of each FG meeting was approximately 50 minutes.

In FG discussions, five main themes were highlighted referring to patient activation via MEDs to seek information on chronic conditions and medications. These themes pertained to motives for seeking information on chronic conditions and medications via MEDs, difficulties and obstacles to seeking information on chronic conditions and medications via MEDs, the overall activation level of patients, the impact of MEDs in activating patients to seek health-related information, and the role of health professionals in facilitating the use of MEDs to enhance patient activation toward seeking health-related information. The themes have been described in more detail below.

### Motives for Seeking Information on Chronic Conditions and Medications via MEDs

For most students and health care professionals, the internet was the main source of information on chronic conditions and medication, whereas health care professionals were the main source of information for most patients. Key identified motives for using MEDs to seek health-related information were using time efficiently, reducing information ambiguity, and getting the most up-to-date medication information. The subthemes with corresponding comments are shown in Textbox 1.

**Theme 1—summary of comments corresponding to each of the subthemes.**

**Detailed Medication Information (+++)**

- “I use the applications mostly for medicines, about the effect of a herb, the action mechanism and the contraindications.” [student]
- “We use MED more about drugs than for diseases because it is a problem to find information and read about diseases on Google.” [health professional]
- “Yes, I even use Facebook. If there is any diet to lose weight, I use it. Even when I receive medication information, I get informed, and when I go to the doctor, I ask him about what I have read, without the doctor’s advice I do not use anything.” [patient]

**Ease and Time Efficiency of Information Access (++)**

- “I think the reason why we use phones, or the Internet is often the time...you will immediately have thousands of publications or links that send you directly to the requested information. The time of searching for a particular problem is shortened, so it is a way of searching much faster than by searching in books.” [student]
- “The use of MED relates to the comfort offered to find information at any time and in any place.” [health professional]
- “We search drugs (online) because it is faster.” [patient]

**Reducing Information Ambiguity (++)**

- “Yes, it has become essential to have a mobile with us. In case I encounter ambiguity...” [student]
- “Yes, we use MED, but it is a bit of a problem to open the phone directly with the patient; then the patient perceives us as we do not know things, but we tell them that, eg, a drug could have 50 or 60 commercial names...” [health professional]

**New Drugs on the Market (++)**

- “...when I hear a new drug name, I search it at least to have the basic information.” [student]
- “We use MED for health and medicines, but mostly to get information about a certain drug that comes out in the market.” [health professional]

**Getting Up-to-Date Information (++)**

- “...information via mobile devices, gets the information fast and uses information that is more updated than books.” [student]
- “...the reasons are to be updated for a certain new medication, to recall and recapture things that could have gone through in the second plan...” [health professional]

**Wider Range of Information (++)**

- “The main motive of looking for a drug is the interest in knowing more drugs because the basic literature is not sufficient.” [student]
- “Internet search through MED provides a wider range of information, all areas are there.” [health professional]

**Queries From Other Family Members or Society (+)**

- “Usually a certain medical condition that I have or someone in the family does, this is the key to pushing me to research, then also for faculty issues if I need something...” [student]

**Medical Condition (++)**

- “On internet you can read something superficially, for illnesses we rather read in books.” [health professional]
- “Our medical condition pushes us to search for information.” [patient]

### Difficulties and Obstacles to Seeking Information on Chronic Conditions and Medications via MEDs

Unlike participants from patient-based FGs who expressed confidence in using MEDs for information retrieval, students and health professionals reported difficulties and obstacles while seeking health and medication information. However, it should be noted that many participants in patient-based FGs did not use MEDs for information searches specifically related to health and medications. The following key difficulties were identified: lack of accurate information in the native language, limited access to the internet, and access to incorrect information. The subthemes with the illustrated comments are shown in Textbox 2.

**Theme 2—summary of comments corresponding to each of the subthemes.**

**Lack of Accurate Information in the Native Language (++)**

- “If the literature was in Albanian, it might have been much easier to understand, but there are data that are not electronically in Albanian, so in other languages it is more difficult to understand.” [student]
- “There is not much information in our [Albanian] language.” [health professional]

**Inability to Access Scientific Journals Due to Subscriptions (+)**

- “The problem is the inability to access publications. In many cases, if we want to read any publication, we read only the abstract.” [student]

**Inability to Access the Internet (+++)**

- “Another obstacle I see is access, connection to the Internet. There are quite accurate apps, but you cannot access them offline.” [student]
- “Another difficulty is the Internet access to our clinics, when we spent our mobile data then our internet access ends.” [health professional]
- “I do not have an internet connection.” [patient]

**Access to Incorrect Information (++)**

- “...if we look for information in electronic books, then I do not see any difficulty, but if I search for information across different web pages, then it could be incorrect.” [student]
- “There is information, but when it comes to different websites, you can read what people who are not competent for that matter have also written.” [health professional]

**Overcoming Obstacles in Seeking Information on Health and Medication (++)**

- “Initially, it would be good to have English language knowledge as most of the accurate and up-to-date information is in English, and this would help us to research more.” [student]
- “Free internet access should be provided in all clinics of UCCK.” [health professional]
- “We should have consultations with pharmacists of the clinics more often because we hardly see them. In this way, we always have to find the information by ourselves, to research it online or to consult with other doctors.” [health professional]

### The Overall Activation Level of Patients

All participants supported the approach that, in general, it is the patients’ responsibility to actively engage in their health management and, therefore, achieve a higher level of activation. This was also the case for the use of MEDs to facilitate patient activation. According to them, the physician’s responsibility is in diagnosing and prescribing, whereas before and after this, it depends on the patient’s behaviors. However, some patients declared that they were not the only ones responsible for actively engaging in their health management. They see themselves and the physician at the same level of responsibility. The subthemes and related comments are listed in Textbox 3.

**Theme 3—summary of comments that correspond to each of the subthemes.**

**Self-care Suggesting a High Level of Activation (+++)**

- “The patient is responsible for his/her health, the doctors are also [responsible], but secondary [compared to the patient]. I think that the patient is more responsible for their own health.” [student]
- “Responsibility is of the doctor when prescribing the drug, but the patient must adhere to the doctor’s counsel. As far as I prescribe metformin and the patient eats baklava [sweets], then it is no longer my responsibility.” [health professional]
- “For your health, you have to be responsible. If you are not responsible for yourself, then the doctor cannot be either.” [patient]
- “Much depends on the patient because the doctor cannot go to the patient’s home and take care of him.” [patient]

**Self-care Suggesting a Low Level of Activation (+)**

- “We face a lot of health-related neglect by patients themselves. It is unimaginable how little we care about ourselves; we care more about our cars...” [health professional]
- “Together, if you do not help the doctor, he finds it difficult [to manage your health].” [patient]

### The Impact of MEDs in Activating Patients to Seek Health-Related Information

The MEDs’ impact on patients was related primarily to their empowerment by facilitating patient activation to seek health-related information. The impact was considered positive if the information was searched adequately and access to accurate and credible information was provided. However, a potential disadvantage to using MEDs from the patients’ perspective, was the possibility of bypassing the physician’s visit. Health professionals suggested that when patients are more informed about their health condition, this facilitates health professionals’ work because of better patient understanding and communication level. To ensure the overall positive impact of MEDs, health professionals suggested the option of providing patients with access to credible health information. Furthermore, patients can be given access to websites with health professionals available to respond to their queries. Information uncertainty and the possibility of information misinterpretation were emphasized to be dangerous. Considering this, it was suggested that patients be informed but not decide about their health based solely on the information they find on the internet. The related subthemes and corresponding comments are listed in Textbox 4.

**Theme 4—summary of comments corresponding to each of the subthemes.**

**Positive Impact of Mobile Electronic Devices in Facilitating Activation in Information Seeking (+++)**

- “MED certainly facilitate the interest in seeking information on health and medicines...” [student]
- “It would be definitely helpful to use MED for health-related information.” [health professional]
- “They [MED] have an impact because everything that interests you can be found there, medicines, food, whatever you want.” [patient]

**Impact of Mobile Electronic Devices in the Patient-Physician Relationship (+++)**

- “Now for the doctor, it has become a bit harder because patients now are [easily] informed about certain types of illnesses, and doctors need to be more careful not to go directly to the diagnosis and medication.” [student]
- “When patients have access to this information, their rapport with doctors is changing a lot, and somehow it is thought that the doctor is replaceable because of technology...” [student]
- “If the patient gets that information from a site or trusted apps, it’s pretty good that when you are in a 24-hour shift, you have 60 to 70 patients within a day, you cannot explain a lot to everyone, and if the patient would be appropriately informed it would be much better and easier.” [health professional]

**Potential Negative Impact of Mobile Electronic Devices in Patients (+++)**

- “Inaccurate online information in patients who have no health information can lead to a worse condition due to the stress they create...We should not replace the doctor with the information that is on the Internet.” [student]
- “The patients have never read more, but also, they have never read nonsense things more. It is good to read, but not so that in every portal is the fluid which heals the heart diseases, the fluid that heals the kidney disease...” [health professional]
- “If I used the internet, I would be schizophrenic. I do not object to the technique, but it is very wide.” [patient]
- “The use of MED for information is a convenience, but it is not a security, so first we need to consult with the relevant doctor.” [student]

### The Role of Health Professionals in Facilitating the Use of MEDs to Enhance Patient Activation Toward Seeking Health-Related Information

Students considered that health professionals’ role in providing information on health and medications is irreplaceable, and according to them, no matter what information can be obtained via the internet, the final source should be health professionals. According to students, there is currently a lack of time management by health professionals during patient contact, causing a lack of health information provision. Health professionals also see their role as a major responsibility but are nevertheless deficient because of the lack of available time. Patients also supported the importance and primary role of health professionals in providing information on health and medications. Regarding the role of health professionals in assisting with the use of MEDs, students suggested that patients should be referred to reliable databases or websites where they could be accurately informed, so the reading of unconfirmed scientific information would be prevented. Health professionals suggested that if they were more active in this role, it could have a significant impact, but this is not the case. Patients considered that if they were instructed to read about their health or medication via MEDs, they would do it and think it would positively affect their health. Subthemes with the comments of participants are shown in Textbox 5.

**Theme 5—summary of comments corresponding to each of the subthemes.**

**Health Professionals Have a Key Role in Providing Health Information (+++)**

- “Despite receiving information through the internet, the main and accurate source should be the doctor.” [student]
- “Informing the client about his/her condition for getting different medication should be done primarily by each health worker.” [health professional]
- “Health professionals have the main role [ie, in providing health information].” [patient]

**Health Professionals' Recommendation on Seeking Health-Related Information Through Mobile Electronic Devices (+++)**

- “It would have been good to inform patients in this regard, but I think very few do. I think it would be much better if you visit a doctor and they help you with using an app that will help you in the future.” [student]
- “I try to give the patient’s family members information on a certain drug, and I recommend them to read on the Internet what we are prescribing.” [health professional]
- “They accomplish this role very well; they tell us to read.” [patient]

**Deficient Role of Health Professionals in Regard to Assisting Patients With Mobile Electronic Devices Used for Health Information (+++)**

- “They have a very important role, but I think they do not use it...they do not give the [exact] information, because of time or other engagements. I think it would have been good to inform patients in this regard [ie, using apps, reading health and medications information through MED], but I think very few do so.” [student]
- “I do not believe it would be functional if a doctor recommends something to the patient to read...” [health professional]
- “It would have been very good if they would have assisted us in this aspect; I would have read and done so.” [patient]

## Discussion

### Principal Findings

This study explored the impact of MEDs on activating patients to seek information on chronic conditions and medications. Data were collected from students, health professionals, and patients. The findings of this study suggest that MEDs could help activate patients to seek information on health and medications. MEDs provide quick access to the information required and provide a large amount of information, which could facilitate patient activation to seek health-related information. Furthermore, a previous observational study showed that mHealth apps were most useful for obtaining general health and physical activity and nutrition information, finding no significant difference in mHealth app’ use on patients’ socioeconomic status [21]. Therefore, the potential role of mHealth apps is empowering patients, especially those with lower socioeconomic status, to improve their health outcomes. Considering that there is evidence that supports the effectiveness of mHealth technologies in clinical outcomes and patient-centered care outcomes such as patient satisfaction and patient engagement [22], this study’s findings are especially important as they further delineate the role of MEDs in facilitating patient activation toward seeking health-related information.

The findings of this study suggest a mutual acceptance of MEDs in patient activation to seek health-related information from patients, health professionals, and future health professionals. However, there were different reasons and approaches for all three subsamples when using the MEDs. Students’ motives for seeking health and medication information through MEDs included easy and quick access and the need for constant information updates that assist them in their studies. Technological barriers were identified and were consistent with previous studies [23], suggesting the need for improvement in access to library-licensed mobile resources. Health professionals seek information through MEDs mainly on new drugs while maintaining a preference for books when searching for information on diseases. This is particularly the case with brand drug names, which may be unknown to some health care staff. In addition, the findings of this study suggest that MEDs in general and mobile apps, in particular, may have a positive impact on the work routine of professional health care staff as they enable them to make quicker decisions with a lower degree of error, thus improving practice efficiency and management of clinical cases. These results are consistent with the findings of previous studies [24,25]. In addition to requiring information on their medications, patients were also interested in knowing more about complementary medications, nutritionally advantageous diets, and healthy lifestyles in general. This is because MEDs provide access to vast amounts of information and a source of unlimited information. Therefore, although findings from all FGs suggest a potential positive role of MEDs in patient activation in seeking health-related information, there were different motives and barriers for each of the study subsamples.

Patients using MEDs and receiving information on their health conditions felt more empowered in managing their health and suggested that higher activation levels could be achieved via the use of MEDs. In contrast, patients who did not use MEDs indicated a lower level of activation and difficulty in understanding the actions required to maintain a healthy lifestyle. However, it was interesting to note that none of the patients identified the existence of nonreliable health-related web-based information as an issue when using MEDs. Therefore, it enhances the need for health professionals to discuss the validity of web-based information that patients may consult and apply to their health.

In addition, although patients are getting more involved in their health care decisions, the proportion who are willing to take serious action and change their health behaviors is still in the minority. It is thus helpful to know which types of patients might become more actively engaged and apt to take such behaviors, and therefore the mechanisms that might be used to aid them and other patients [26]. Although MEDs could facilitate patient activation in seeking health-related information for some patients, the role of MEDs might be vague for some others. Therefore, besides evaluating the patient activation level, assessing the stage of change in the transtheoretical model (TTM) might help determine for which patients MEDs could affect patient activation in seeking health-related information.

Additional research might consider using the TTM, an integrative biopsychosocial model aimed at predicting one’s likelihood of behavior change [27]. Using the TTM can help discern where patients are in their contemplation to engage and use mobile devices and technology for their health, and thus might be used in concert with the results of our study to design educational interventions and communication with patients.

This study suggests that health professionals can influence the activation of patients to seek health-related information using MEDs by assisting them in the use of MEDs to search for information, which would be an additional motive for patients. This guidance and assistance could occur during regular check-ups that patients with chronic conditions have. During this time, health care professionals could advise using certain credible websites to require health-related information and even try to conduct a web search related to the patient’s specific concerns. This assistance would facilitate patients’ interest in using MEDs for seeking information on health and medications. However, the health professionals’ use of MEDs might influence their ability to support and guide patients in their use related to seeking health-related information. Evidence indicates that adequate training of health professionals on this matter would influence health education and improve the population’s overall health on primary and secondary prevention bases [28]. However, it is promising that future health professionals tend to highly use MEDs, positing them in a better empowerment position to assist and advise their patients regarding seeking health-related information on the web than older cohorts of health professionals.

Furthermore, it is interesting to note that even patients who did not use MEDs for information on health and medications suggested that they would do so if health professionals would recommend specific information websites or mobile apps. The reliability of web-based health information remains a concern [29], and the quality of health information accessed by patients remains unevaluated [30]. Thus, as students and health professionals would recognize a valid data source, it would be questionable for patients with different training and backgrounds. In addition, evidence indicates that age differences play a vital role in credibility judgments among patients seeking health information on the web, showing that older adults have a higher tendency to passively accept web-based information compared with younger adults [31], thus suggesting a need for different training approaches to these populations. Finally, research indicates that the only predictor of mHealth use for self-management was patient information technology skills [32]. Therefore, health care professionals should advise all patients about MEDs, regardless of their age.

### Study Limitations

This study had several limitations. First, the study lacks wider representativeness, as it was conducted in one city of Kosovo, and it did not include a wider range of participants who could be potential users of MEDs. This limitation can be considered minimal in the results obtained because participants originated from various parts of Kosovo, and a saturation point in terms of themes and subthemes was achieved even across diverse groups of individuals. The generalizability of this study’s results is questionable because of the sample size and sample characteristics. However, considering that in 2019 the global internet access rate was 51.4%, and it was estimated that 86.7% of the population in developed countries had internet access [33], data from a study in 2017 showed that 88.5% of households in Kosovo had internet access [34]. Therefore, this study’s findings would be more applicable in countries with similar internet access coverage. However, the qualitative *research* goal is not the generalizability as much as it is the generation of rich and contextualized understanding of unexplored phenomena [35].

In addition, FGs with health professionals consisted of specialists in various fields of medicine and nurses. The diversity of health professionals in FGs may have facilitated exploring different perspectives, although segmentation of these FG participants could have facilitated comparative data analysis. However, it has been previously reported that homogeneous groups of participants in FG meetings can capitalize on the shared experiences of participants [36]; thus, this approach was used to conduct separate FGs with students, health professionals, and patients. Finally, the FGs were conducted with participants in Kosovo, who were almost entirely of ethnic Albanian descent. As qualitative research, there was no instrumentation to translate directly; however, the concepts and theories from which the FG guide was constructed had their basis in the English language literature. Furthermore, this potential limitation should also be considered in lieu of the fact that regardless of location and language used, access to the internet, MEDs, and mobile apps has increased significantly worldwide; therefore, patients and health professionals are expected to exhibit similar behaviors when adopting technology. In this regard, it may be worth emphasizing that Kosovo is known to have the highest levels of household internet access in the world [37]. Nonetheless, we believe there is a unique internet- and media-related characteristic of our sample that may affect the use of MEDs to navigate health-related information. This uniqueness is derived from the fact that the Kosovo population has strong family and sociocultural ties with its large diaspora living overseas and with whom there is a high reliance on MEDs to exchange information on a regular basis. In this environment, MEDs users in Kosovo would be exposed to cross-cultural experiences derived from various societies that the Kosovo diaspora has influenced globally, which in turn may also have an impact on how they assess and interpret information. This characteristic of our sample as well as of similar populations groups merits further research to better understand the implications on navigation via MEDs and the use of health-related information.

This study provides data referring to some of the basic motives for using MEDs for information search on health and medications and suggests the need for a change in health professionals’ approach to assist using MEDs to facilitate patient activation in this regard. There is a need for further research into the clinical and economic impact of using MEDs in facilitating patients’ activation to seek information on their chronic conditions and medications.

### Conclusions

This study suggests that MEDs might help facilitate patients’ activation to seek information on chronic conditions and medications. The motives for searching for information through MEDs related to the activation of patients have been identified. The findings suggest that health professionals’ roles should be reconsidered to include additional assistance to their patients in using MEDs, specifically in recommending valid and trustworthy websites or mobile apps to search for information on chronic conditions and medications.

## Abbreviations

FG: focus group
MED: mobile electronic device
mHealth: mobile health
TTM: transtheoretical model
UCCK: University Clinical Center of Kosovo

## References

[1] Hibbard J, Gilburt H (2014). Supporting people to manage their health: An introduction to patient activation. Kings Fund.

[2] Charmel PA, Frampton SB (2008). Building the business case for patient-centered care. Healthcare Financial Management Association.

[3] Kilo CM, Wasson JH (2010). Practice redesign and the patient-centered medical home: history, promises, and challenges. Health Aff (Millwood).

[4] Greene J, Hibbard JH (2012). Why does patient activation matter? An examination of the relationships between patient activation and health-related outcomes. J Gen Intern Med.

[5] Hibbard JH, Greene J, Overton V (2013). Patients with lower activation associated with higher costs; delivery systems should know their patients' 'scores'. Health Aff (Millwood).

[6] Smith SG, Pandit A, Rush SR, Wolf MS, Simon C (2014). The association between patient activation and accessing online health information: results from a national survey of US adults. Health Expect.

[7] Hibbard JH, Cunningham PJ (2008). How engaged are consumers in their health and health care, and why does it matter?. Res Brief.

[8] Martin T (2012). Assessing mHealth: opportunities and barriers to patient engagement. J Health Care Poor Underserved.

[9] Siau K, Shen Z (2006). Mobile healthcare informatics. Med Inform Internet Med.

[10] O'Leary KJ, Lohman ME, Culver E, Killarney A, Randy SG, Liebovitz DM (2015). The effect of tablet computers with a mobile patient portal application on hospitalized patients' knowledge and activation. J Am Med Inform Assoc.

[11] Choi A, Lovett AW, Kang J, Lee K, Choi L (2015). Mobile applications to improve medication adherence: existing apps, quality of life and future directions. Adv Pharmacol Pharm.

[12] Moura J, Almeida AM, Roque F, Figueiras A, Herdeiro MT (2021). A mobile app to support clinical diagnosis of upper respiratory problems (eHealthResp): co-design approach. J Med Internet Res.

[13] Pecina JL, Wyatt KD, Comfere NI, Bernard ME, North F (2017). Uses of mobile device digital photography of dermatologic conditions in primary care. JMIR Mhealth Uhealth.

[14] Martinez-Millana A, Jarones E, Fernandez-Llatas C, Hartvigsen G, Traver V (2018). App features for type 1 diabetes support and patient empowerment: systematic literature review and benchmark comparison. JMIR Mhealth Uhealth.

[15] Kaboli PJ, Howren MB, Ishani A, Carter B, Christensen AJ, Vander Weg MW (2018). Efficacy of patient activation interventions with or without financial incentives to promote prescribing of thiazides and hypertension control: a randomized clinical trial. JAMA Netw Open.

[16] Silver L, Smith A, Johnson C, Jiang J, Anderson M, Rainie L (2019). Mobile connectivity in emerging economies. Pew Research Center.

[17] Morgan DL (1996). Focus groups. Annu Rev Sociol.

[18] Stewart D, Shamdasani P (2014). Focus Groups Theory and Practice.

[19] Williams M, Moser T (2019). The art of coding and thematic exploration in qualitative research. International Management Review.

[20] Hoti K, Hughes J, Sunderland B (2012). Medication supply to residential aged care facilities in Western Australia using a centralized medication chart to replace prescriptions. BMC Geriatr.

[21] Ramirez V, Johnson E, Gonzalez C, Ramirez V, Rubino B, Rossetti G (2016). Assessing the use of mobile health technology by patients: an observational study in primary care clinics. JMIR Mhealth Uhealth.

[22] Bruce CR, Harrison P, Nisar T, Giammattei C, Tan NM, Bliven C, Shallcross J, Khleif A, Tran N, Kelkar S, Tobias N, Chavez AE, Rivera D, Leong A, Romano A, Desai SN, Sol JR, Gutierrez K, Rappel C, Haas E, Zheng F, Park KJ, Jones S, Barach P, Schwartz R (2020). Assessing the impact of patient-facing mobile health technology on patient outcomes: retrospective observational Cohort study. JMIR Mhealth Uhealth.

[23] Boruff JT, Storie D (2014). Mobile devices in medicine: a survey of how medical students, residents, and faculty use smartphones and other mobile devices to find information. J Med Libr Assoc.

[24] Ventola CL (2014). Mobile devices and apps for health care professionals: uses and benefits. Pharm Ther.

[25] Siebert JN, Lacroix L, Cantais A, Manzano S, Ehrler F (2020). The impact of a tablet app on adherence to american heart association guidelines during simulated pediatric cardiopulmonary resuscitation: randomized controlled trial. J Med Internet Res.

[26] Prochaska JO (2008). Decision making in the transtheoretical model of behavior change. Med Decis Making.

[27] Prochaska JO, Velicer WF (1997). The transtheoretical model of health behavior change. Am J Health Promot.

[28] Bert F, Giacometti M, Gualano MR, Siliquini R (2014). Smartphones and health promotion: a review of the evidence. J Med Syst.

[29] Epstein HB (2017). Cyberchondriacs. J Hosp Librariansh.

[30] Diaz JA, Griffith RA, Ng JJ, Reinert SE, Friedmann PD, Moulton AW (2002). Patients' use of the internet for medical information. J Gen Intern Med.

[31] Liao QV, Fu W (2014). Age differences in credibility judgments of online health information. ACM Trans Comput-Hum Interact.

[32] Jiang J, Zhu Q, Zheng Y, Zhu Y, Li Y, Huo Y (2019). Perceptions and acceptance of mHealth in patients with cardiovascular diseases: a cross-sectional study. JMIR Mhealth Uhealth.

[33] (2020). Global internet penetration 2020. Statista.

[34] (2017). Results of the survey on use of information and communication technology. Kosovo Agency of Statistics - Series 5: Social Statistics.

[35] Polit DF, Beck CT (2010). Generalization in quantitative and qualitative research: myths and strategies. Int J Nurs Stud.

[36] Kitzinger J (1995). Qualitative research. Introducing focus groups. Br Med J.

[37] (2020). Digital economy and society statistics - households and individuals. Eurostat.

